# Identifying drivers of food insecurity through linked data- the Priority Places for Food Index

**DOI:** 10.23889/ijpds.v8i3.2277

**Published:** 2023-09-11

**Authors:** Francesca Pontin, Peter Baudains, Emily Ennis, Michelle Morris

**Affiliations:** 1 Consumer Data Research Centre, University of Leeds; 2 School of Food Science and Nutrition, University of Leeds

## Introduction & Background

15.5% of all UK households are food insecure; either unable to afford to eat, skipping meals or reducing meal sizes despite being hungry. Drivers of food insecurity include both access to and affordability of food, with those most in need often left unable to access healthy and affordable food. Taking a place-based approach to understand the drivers of food insecurity allows for targeted support from government, third sector and private organisations to mitigate growing food insecurity in the UK.

## Objectives & Approach

This research presents the methodology for the co-production and construction of the Priority Places for Food Index (PPFI) and supporting dashboard, co-designed by the Consumer Data Research Centre (CDRC) and consumer champions Which? in response to the 'cost-of-living crisis'.

The PPFI equally weights measures of access to affordable food and indicators of barriers to affording food across seven domains; Proximity to supermarket retail facilities, Accessibility of supermarket retail facilities, Access to online deliveries, Proximity to non-supermarket food provision, Socio-economic barriers, Fuel Poverty and Family Food for support. The PPFI uses open data combing traditional census data metrics, with government data (e.g., Healthy start voucher and free-school meals uptake), digital footprints data (web-scraped delivery addresses and food bank item request data) and scaled-survey data (fuel poverty, propensity to shop online).

## Relevance to Digital Footprints

Digital footprint data can complement traditional data sources to provide a more nuanced view of health inequalities. These data are typically less timely to collect than traditional data collection methods (census, survey) allowing a more reactive response to emergent issues such as the cost-of-living crisis.

## Results

The PPFI interactive map and underlying data have been published via the CDRC https://priorityplaces.cdrc.ac.uk/.

## Conclusions & Implications

We demonstrate the value of data linkage across individual and population level data to provide localised insight into food insecurity and identify where digital footprints data can improve gaps in the current evidence base. We also reflect on the value of co-production and stakeholder engagement in creating a policy ready interactive map which has facilitated the lobbying of targeted practical support and policy change to address food insecurity.

